# Risks of ophthalmic disorders in patients with systemic lupus erythematosus – a secondary cohort analysis of population-based claims data

**DOI:** 10.1186/s12886-020-01360-w

**Published:** 2020-03-11

**Authors:** Chun-Shuo Hsu, Chia-Wen Hsu, Ming-Chi Lu, Malcolm Koo

**Affiliations:** 1 Division of Obstetrics and Gynecology, Dalin Tzu Chi Hospital, Buddhist Tzu Chi Medical Foundation, Chiayi, Taiwan; 2 School of Medicine, Tzu Chi University, Hualien, Taiwan; 3 Department of Medical Research, Dalin Tzu Chi Hospital, Buddhist Tzu Chi Medical Foundation, Chiayi, Taiwan; 4 Division of Allergy, Immunology and Rheumatology, Dalin Tzu Chi Hospital, Buddhist Tzu Chi Medical Foundation, No. 2, Minsheng Road, Dalin, Chiayi, 62247 Taiwan; 5 Graduate Institute of Long-term Care, Tzu Chi University of Science and Technology, Hualien, Taiwan; 6 Dalla Lana School of Public Health, University of Toronto, Toronto, Ontario Canada

**Keywords:** Systemic lupus erythematosus, Ophthalmic disorders, Dry eye disease, Cataracts, Glaucoma

## Abstract

**Background:**

Systemic lupus erythematosus (SLE) can directly affect various part of the ocular system, but there was no comprehensive analysis of ophthalmic disorders of patients with SLE using population-based data. The aim of this study was to investigate the frequency and prevalence of ophthalmic disorders for ophthalmologist visits in adult patients with SLE and to evaluate the risk of dry eye syndrome, cataracts, glaucoma, episcleritis and scleritis, and retinal vascular occlusion in these patients.

**Methods:**

The Taiwan’s National Health Insurance Research Database was used to assemble a SLE cohort consisting of newly diagnosed SLE between 2000 and 2012. A comparison cohort was also sampled from the same database and it consisted of 10 patients without SLE for each patient with SLE, based on frequency matching for sex, five-year age interval, and index year. Both cohorts were followed until either the study outcomes have occurred or the end of the follow-up period.

**Results:**

Patients with SLE (*n* = 521) exhibited a significantly higher prevalence (68.1% vs. 60.5%, *P* = 0.001) and frequency (median 5.51 vs. 1.71 per 10 years, *P* <  0.001) for outpatient ophthalmologist visits compared with patients without SLE. The risk of dry eye syndrome (adjusted incidence rate ratio [IRR] 4.45, *P* <  0.001), cataracts (adjusted IRR 3.18, *P* <  0.001), and glaucoma (adjusted IRR 2.23, *P* = 0.002) were significantly higher in patients with SLE. In addition, the risk of several SLE related ophthalmic disorders, including episcleritis and scleritis (adjusted IRR 6.11, *P* <  0.001) and retinal vascular occlusion (adjusted IRR 3.81, *P* = 0.023) were significantly higher in patients with SLE.

**Conclusions:**

The increased risk of dry eye syndrome, cataracts, glaucoma, episcleritis and scleritis, and retinal vascular occlusion in patients with SLE deserves vigilance.

## Background

Systemic lupus erythematosus (SLE) is a chronic systemic autoimmune disease that is associated with considerable morbidity and mortality [1]. SLE typically develops in childbearing age women, with a female to male ratio of approximately 9:1. Multiple body organs and systems, such as the kidney, brain, lung, and the hematologic or musculoskeletal systems can be affected. In addition, SLE can directly attack the retina, lacrimal gland, choroid, optical nerve, and even the episclera and sclera of the ocular system [2, 3]. It may also affect the ocular system indirectly through the adverse effects of long-term administration of certain medications for SLE [4] and infection related to impaired immunity [5].

Although several studies have addressed the ocular manifestations of SLE [6–9], there were no comprehensive analyses of ophthalmic disorders of patients with SLE using population-based data. Therefore, we used the Taiwan’s National Health Insurance Research Database (NHIRD) [10], which is a nationwide, population-based database containing comprehensive medical service utilization records of over 99% of Taiwan’s 23-million population, to compare the frequency of outpatient ophthalmologist visits between patients with and without SLE. We also explored common diagnoses of eye disorders for ophthalmic clinic visits in patients with SLE. Furthermore, we assessed the incidence and risk of several important ophthalmic disorders in patients with SLE.

## Methods

### The SLE cohort and a comparison cohort

This study used a retrospective cohort design based on the claim data from the Taiwan’s NHIRD. The study protocol was approved by the institutional review board of the Dalin Tzu Chi Hospital, Buddhist Tzu Chi Medical Foundation, Chiayi, Taiwan (No. B10104020). The requirement for obtaining informed consent from the patients was waived by the institutional review board because the NHIRD contain deidentified information.

**Fig. 1:**
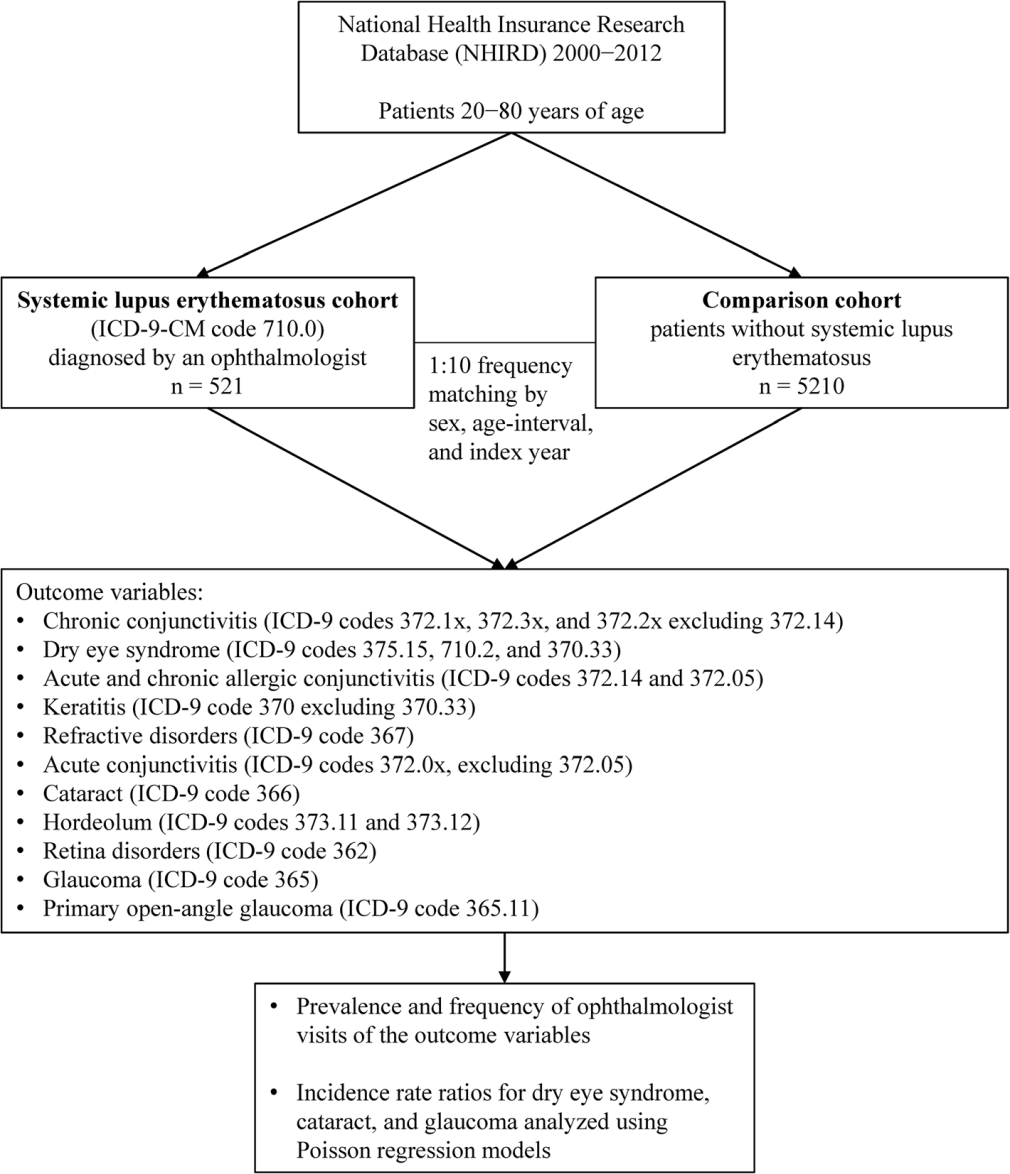
Study flow chart

Patients with SLE were identified from the 2000–2012 catastrophic illness datafile, which is a subset of the NHIRD, based on the International Classification of Diseases, Ninth revision, clinical modification (ICD-9-CM) code 710.0. In Taiwan, SLE is officially considered as a catastrophic illness, and patients with SLE are eligible to apply for a certificate from the National Health Insurance Administration. The certificate is issued to patients after their medical records and serological reports have been reviewed by the National Health Insurance Administration based on the 1997 American College of Rheumatology revised criteria for the classification of SLE [11]. Successful applicants are exempted from their SLE-related health care copayment fee. In this study, the date of the application of the catastrophic illness certificate was defined as the index date for the SLE patients. Patients aged under 20 (the legal age of adulthood in Taiwan) or over 80 years on the index date were excluded. The upper age limit was set at 80 years because the increased prevalence of disabilities among these individuals is likely to limit their visits to ophthalmology clinics. Figure 1 shows the flowchart of the enrollment of the study cohort.

A comparison cohort was random sampled from the outpatient datafile of the 2000 Longitudinal Health Insurance Database (LHID 2000) with claim records between January 1, 2000 and December 31, 2012. The LHID 2000 is a subfile of the NHIRD containing health claim data for one million beneficiaries randomly sampled from all enrollees of the NHIRD in 2000. For each patient with SLE, 10 patients were selected, based on frequency matching for sex, 5-year age interval, and index year.

### Identification of frequency of ophthalmic disorders

Both the SLE cohort and the comparison cohort were followed until a diagnosis of the ophthalmic outcomes in this study by an ophthalmologist or the end of the follow-up period. The latter was defined as the last date of an outpatient visit for each patient. We reviewed all the ophthalmic disorders of patients with SLE using ICD-9-CM codes and selected 10 ophthalmic disorders based on the number of ophthalmologist outpatient visits. The 10 ophthalmic disorders assessed were (1) chronic conjunctivitis excluding allergic conjunctivitis (ICD-9-CM codes 372.1x, 372.3x, and 372.2x excluding 372.14), (2) dry eye syndrome (ICD-9-CM codes 375.15, 710.2, and 370.33), (3) acute and chronic allergic conjunctivitis (ICD-9-CM codes 372.14 and 372.05), (4) keratitis (ICD-9-CM code 370 excluding 370.33), (5) refractive disorders (ICD-9-CM code 367), (6) acute conjunctivitis (ICD-9-CM codes 372.0x, excluding 372.05), (7) cataracts (ICD-9-CM code 366), (8) hordeolum (ICD-9-CM codes 373.11 and 373.12), (9) retina disorders (ICD-9-CM code 362), and (10) glaucoma (ICD-9-CM code 365). Furthermore, primary open-angle glaucoma (POAG) (ICD-9-CM code 365.11) was also assessed because neuroendocrine-immune abnormalities have been hypothesized to play an important role in the optic neuropathy of POAG [12].

In Taiwan, most of the ophthalmic disorders are diagnosed based on a combination of clinical assessments. Patients seen in ophthalmic clinics typically undergo a medical history review followed by an ophthalmologic examination, including slit lamp examination, applanation tonometry, confrontation visual field examination, testing for eye movement, refraction examination via both autorefraction and manifest refraction, Schirmer’s test, and dilated fundus examination. In addition, dry eye syndrome is generally diagnosed according to the diagnostic criteria proposed by the Japan Dry Eye Society [13].

To restrict the identification of only newly diagnosed ophthalmic disorders, patients who had diagnosed with ophthalmic disorders before the index date were excluded. Since the NHIRD was assembled since 1996, and our study period started from 2000, we had an interval of at least 4 years for excluding non-eligible patients, that is, those who had one of the 10 ophthalmic disorders prior to the index date.

The incidence and risk of several important ophthalmic disorders, including dry eye syndrome, cataracts, glaucoma, episcleritis and scleritis (ICD-9-CM code 379.0x), retinal vascular occlusion (ICD-9-CM code 362.3x), retinal vasculitis (ICD-9-CM code 362.18), and neovascular glaucoma (ICD-9-CM code 365.63) in patients with SLE were calculated and compared with the comparison cohort.

### Statistical analysis

The basic characteristics between the SLE and the comparison cohorts were compared using Chi-square test, t-test, or Mann-Whitney U-test, as appropriate. The prevalence and frequency of the 10 ophthalmic disorders between the SLE cohort and the comparison cohort were compared using Chi-square test and Mann-Whitney U-test, respectively. Since the data for the number of visits were not normally distributed, they are presented as median and interquartile range, in addition to mean and standard deviation.

In addition, incidence rates per 1000 person-years were calculated for the SLE cohort and the comparison cohort. Poisson regression models (i.e., generalized linear models with a Poisson log-linear link function and person-years as the offset variable) were used to calculate incidence rate ratios (IRR) for the outcome variables, with or without adjusting for the potential confounding effect of age, sex, socioeconomic status, and geographical region. Additional subgroup analyses were also performed with stratification by sex, and age groups (20–39, 40–59, and > 60 years) when the sample size permitted. All analyses were conducted using IBM SPSS Statistics for Windows, version 24.0 (IBM Corp, Armonk, NY, USA). A two-sided *P* value of < 0.05 was considered statistically significant.

## Results

### Basic characteristics of patients in the SLE and comparison cohorts

The basic characteristics of the 5731 patients in the SLE cohort and the comparison cohort are shown in Table 1. No significant differences were observed between the two groups with respect to sex, age, and geographic region, but the socioeconomic status, which was estimated by the insurance premium level was significantly higher in the SLE cohort.

**Table 1:** Basic characteristics of the systemic lupus erythematosus cohort and comparison cohort (*N* = 5731)

Variable	N (%)				*P*
	systemic lupus erythematosus cohort 521 (9.1)		comparison cohort 5210 (90.9)		
Sex					> 0.999
male	59	(11.3)	590	(11.3)	
female	462	(88.7)	4620	(88.7)	
Age group (years)					> 0.999
20–39	281	(53.9)	2810	(53.9)	
40–59	169	(32.5)	1690	(32.5)	
> 60	71	(13.6)	710	(13.6)	
Mean age (standard deviation), years	41.2	(15.8)	40.2	(15.8)	0.986
Median age (interquartile range), years	38	(28–52)	38	(28–52)	
Socioeconomic status (*n* = 5724)					0.009
low	266	(51.5)	3000	(57.6)	
middle	139	(26.9)	1324	(25.4)	
high	112	(21.7)	883	(17.0)	
Geographic region (*n* = 5558)					0.766
northern	297	(59.5)	3119	(61.7)	
central	93	(18.6)	859	(17.0)	
southern	99	(19.8)	976	(19.3)	
eastern	10	(2.0)	105	(2.1)	

Socioeconomic status was estimated by insurance premiums based on salary. Low: ≤ 19,000 New Taiwan dollars (NT$); middle: 19,001 − 24,000; and high: > 24,000

*P* values were obtained by Chi-square test for categorical variables and t-test or Mann-Whitney U-test for continuous variables, as appropriate

### Frequency of outpatient ophthalmologist visits and frequency of ophthalmic disorders

Patients with SLE had a higher proportion suffering from ophthalmic disorders (68.1% vs. 60.5%; *P* = 0.001) and a higher median frequency of ophthalmologist visits per year compared with the patients in the comparison cohort (5.5 vs. 1.7 per 10 years, *P* <  0.001) (Table 2). Table 2 also shows the ophthalmic disorders assessed in this study. Except for hordeolum, glaucoma, and POAG, the proportion and frequency of ophthalmologist visits for all ophthalmic disorders were significantly higher in the SLE cohort compared with the comparison cohort.

**Table 2:** The prevalence and frequency of ophthalmic disorders in the systemic lupus erythematosus cohort and comparison cohort (*N* = 5731)

Variable	N (%)				*P*
	systemic lupus erythematosus cohort 521 (9.1)		comparison cohort 5210 (90.9)		
**Ophthalmologist visits**					
Prevalence (%)	355	(68.1)	3151	(60.5)	0.001
Number of visits, median (IQR) (/10 years)	5.51	(0–21.28)	1.71	(0–7.02)	< 0.001
Number of visits, mean (SD) (/10 years)	21.15	(42.22)	7.98	(18.50)	
**Chronic conjunctivitis** (excluding chronic allergic conjunctivitis)					
Prevalence (%)	258	(50.5)	1568	(30.1)	< 0.001
Number of visits, median (IQR) (/10 years)	0	(0–3.58)	0	(0–1.07)	< 0.001
Number of visits, mean (SD) (/10 years)	3.81	(8.01)	1.94	(7.30)	
**Dry eye syndrome**					
Prevalence (%)	144	(27.6)	365	(7.0)	< 0.001
Number of visits, median (IQR) (/10 years)	0	(0–0.98)	0	(0–0)	< 0.001
Number of visits, mean (SD) (/10 years)	2.97	(9.65)	0.46	(3.71)	
**Acute and chronic allergic conjunctivitis**					
Prevalence (%)	108	(20.7)	681	(13.1)	< 0.001
Number of visits, median (IQR) (/10 years)	0	(0–0)	0	(0–0)	< 0.001
Number of visits, mean (SD) (/10 years)	0.95	(3.70)	0.51	(3.26)	
**Keratitis (excluding dry eye syndrome)**					
Prevalence (%)	90	(17.3)	437	(8.4)	< 0.001
Number of visits, median (IQR) (/10 years)	0	(0–0)	0	(0–0)	< 0.001
Number of visits, mean (SD) (/10 years)	1.03	(4.50)	0.28	(1.76)	
**Refractive disorders**					
Prevalence (%)	90	(17.3)	315	(6.0)	< 0.001
Number of visits, median (IQR) (/10 years)	0	(0–0)	0	(0–0)	< 0.001
Number of visits, mean (SD) (/10 years)	0.75	(3.28)	0.18	(1.20)	
**Acute conjunctivitis** (excluding acute allergic conjunctivitis)					
Prevalence (%)	88	(16.9)	545	(10.5)	< 0.001
Number of visits, median (IQR) (/10 years)	0	(0–0)	0	(0–0)	< 0.001
Number of visits, mean (SD) (/10 years)	0.52	(1.80)	0.28	(1.97)	
**Cataracts**					
Prevalence (%)	85	(16.3)	431	(8.3)	< 0.001
Number of visits, median (IQR) (/10 years)	0	(0–0)	0	(0–0)	< 0.001
Number of visits, mean (SD) (/10 years)	1.97	(7.05)	0.84	(4.98)	
**Hordeolum**					
Prevalence (%)	73	(14.0)	637	(12.2)	0.238
Number of visits, median (IQR) (/10 years)	0	(0–0)	0	(0–0)	0.224
Number of visits, mean (SD) (/10 years)	0.40	(1.49)	0.31	(1.19)	
**Retina disorder**					
Prevalence (%)	44	(8.4)	202	(3.9)	< 0.001
Number of visits, median (IQR) (/10 years)	0	(0–0)	0	(0–0)	< 0.001
Number of visits, mean (SD) (/10 years)	0.87	(6.32)	0.31	(2.96)	
**Glaucoma**					
Prevalence (%)	20	(3.8)	133	(2.6)	0.083
Number of visits, median (IQR) (/10 years)	0	(0–0)	0	(0–0)	0.085
Number of visits, mean (SD) (/10 years)	0.52	(4.29)	0.61	(6.71)	
**Primary open-angle glaucoma**					
Prevalence (%)	2	(0.4)	27	(0.5)	0.680
Number of visits, median (IQR) (/10 years)	0	(0–0)	0	(0–0)	
Number of visits, mean (SD) (/10 years)	0.07	(1.44)	0.13	(2.42)	0.680

*P* values were obtained by Chi-square test for comparison of prevalence and Mann-Whitney U-test for comparison of medians of number of visits

*IQR* Interquartile range, *SD* Standard deviation

### Risk of developing dry eye syndrome in patients with SLE

The incidence rates and IRRs for developing dry eye syndrome in the SLE cohort and the comparison cohort, with and without stratification by sex or age group are shown in Table 3. Patients in the SLE cohort exhibited a significantly higher risk of developing dry eye syndrome compared with those in the comparison cohort (adjusted IRR 4.45, *P <* 0.001). When the analyses were conducted with stratification by sex, similar magnitudes of IRRs for dry eye syndrome were observed in both male patients (adjusted IRR 4.46, *P* <  0.001) and female patients (adjusted IRR 4.46, *P* <  0.001). In addition, adjusted IRRs for dry eye syndrome were significantly elevated for all three age groups in the SLE cohort compared with the comparison cohort, with the largest magnitude in the 40–59 years group (adjusted IRR 4.89, *P* <  0.001).

**Table 3:** The incidence rate and incidence risk ratio of dry eye syndrome in the systemic lupus erythematosus cohort and comparison cohort (*N* = 5393)

Disorder (ICD-9-CM)		SLE cohort (*n* = 450)			comparison cohort (*n* = 4943)			IRR (95% CI)	Adjusted IRR^a^ (95% CI)
		No. of patient	Person-years	IR	No. of patient	Person-years	IR	*P*	*P*
Dry eye syndrome (375.15, 710.2, 370.33)	Overall	107	2577	41.52	298	32,189	9.26	4.48 (3.60–5.59) < 0.001	4.45 (3.54–5.58) < 0.001
	Sex								
	male	9	259	34.75	29	3472	8.35	4.16 (1.97–8.78) < 0.001	4.46 (2.08–9.58) < 0.001
	female	98	2318	42.28	269	28,717	9.37	4.51 (3.58–5.69) < 0.001	4.45 (3.50–5.65) < 0.001
	Age group (years)								
	20–39	50	1564	31.97	126	18,357	6.86	4.66 (3.37–6.46) < 0.001	4.43 (3.16–6.21) < 0.001
	40–59	43	787	54.64	117	10,618	11.02	4.96 (3.50–7.03) < 0.001	4.89 (3.41–7.01) < 0.001
	> 60	14	226	61.95	55	3214	17.11	3.62 (2.01–6.51) < 0.001	3.50 (1.94–6.34) < 0.001

*CI* Confidence interval, *ICD-9-CM* International Classification of Diseases, Ninth revision, clinical modification, *IR* Incidence rate per 1000 person-years, *IRR* Incidence rate ratio, *n.c*. Not calculable, *SLE* Systemic lupus erythematosus

^a^Adjusted for age, sex, socioeconomic status, and geographic region

### Risk of developing cataracts in patients with SLE

The incidence rates and IRRs for developing cataracts in the SLE cohort and the comparison cohort, with and without stratification by sex and age group are shown in Table 4. Overall, patients in the SLE cohort exhibited a significantly higher risk of developing cataracts compared with the comparison cohort (adjusted IRR 3.18, *P* <  0.001). Both male and female patients with SLE had a significantly increased risk of developing cataracts (adjusted IRR 2.13, *P* = 0.041 and adjusted IRR 3.50, *P* <  0.001, respectively). Moreover, the IRR for the development of cataracts in SLE patients were significantly elevated in all three age groups, with marked increase in the 20–39 years group (adjusted IRR 13.93, *P* <  0.001) compared with those in the comparison cohort.

**Table 4:** The incidence rate and incidence rate ratio of cataracts in the systemic lupus erythematosus cohort and comparison cohort (*N* = 5225)

Disorder (ICD-9-CM)		SLE cohort (*n* = 468)			comparison cohort (*n* = 4757)			IRR (95% CI)	Adjusted IRR^a^ (95% CI)
		No. of patient	Person-years	IR	No. of patient	Person-years	IR	*P*	*P*
Cataracts (366)	Overall	62	2844	21.80	274	31,421	8.72	2.50 (1.90–3.29) < 0.001	3.18 (2.39–4.22) < 0.001
	Sex								
	male	9	235	38.30	61	3060	19.93	1.92 (0.96–3.87) 0.067	2.13 (1.03–4.38) 0.041
	female	53	2609	20.31	213	28,361	7.51	2.70 (2.00–3.65) < 0.001	3.50 (2.56–4.80) < 0.001
	Age group (years)								
	20–39	15		8.26		18,926	0.69	12.03 (5.73–25.28) < 0.001	13.93 (6.49–29.90) < 0.001
	40–59	34	1815	36.29	13	10,804	13.24	2.74 (1.89–3.98) < 0.001	2.78 (1.89–4.09) < 0.001
	> 60	13	937	141.30	143	1692	69.74	2.02 (1.14–3.59) 0.016	2.03 (1.11–3.71) 0.022

*CI* Confidence interval, *ICD-9-CM* International Classification of Diseases, Ninth revision, clinical modification, *IR* Incidence rate per 1000 person-years, *IRR* Incidence rate ratio; *n.c*. Not calculable, *SLE* Systemic lupus erythematosus

^a^Adjusted for age, sex, socioeconomic status, and geographic region

### Risk of developing glaucoma in patients with SLE

The incidence rates and IRRs for developing glaucoma in the SLE cohort and the comparison cohort, with and without stratification by sex and age group are shown in Table 5. Overall, patients in the SLE cohort exhibited a significantly higher risk of developing glaucoma compared with those in the comparison cohort (adjusted IRR 2.23, *P* = 0.002). Both male and female patients with SLE had a significantly increased risk of developing glaucoma (adjusted IRR 4.25, *P* = 0.006, and adjusted IRR 1.87, *P* = 0.038, respectively). In addition, the IRR for glaucoma in patients with SLE were significantly elevated only in the 20–39 years group (adjusted IRR 3.44, *P* = 0.003) compared with those in the comparison cohort.

**Table 5:** The incidence rate and incidence rate ratio of glaucoma in the systemic lupus erythematosus cohort and comparison cohort (*N* = 5593)

Disorder (ICD-9-CM)		SLE cohort (n = 450)			comparison cohort (n = 4943)			IRR (95% CI)	Adjusted IRR^a^ (95% CI)
		No. of patient	Person-years	IR	No. of patient	Person-years	IR	*P*	*P*
Glaucoma (365)	Overall	18	3116	5.78	96	33,351	2.88	2.01 (1.21–3.32) 0.007	2.23 (1.35–3.70) 0.002
	Sex								
	male	5	260	19.23	17	3530	4.82	4.00 (1.47–10.83) 0.006	4.25 (1.52–11.88) 0.006
	female	13	2856	4.55	79	29,821	2.65	1.72 (0.96–3.09) 0.071	1.87 (1.03–3.37) 0.038
	Age group (years)								
	20–39	8	1800	4.44	25	18,875	1.32	3.36 (1.51–7.44) 0.003	3.44 (1.54–7.69) 0.003
	40–59	6	1051	5.71	45	11,183	4.02	1.42 (0.61–3.33) 0.421	1.54 (0.65–3.61) 0.326
	> 60	4	265	15.09	26	3293	7.90	1.91 (0.67–5.48) 0.227	2.14 (0.74–6.20) 0.163

*CI* Confidence interval, *ICD-9-CM* International Classification of Diseases, Ninth revision, clinical modification, *IR* incidence rate per 1000 person-years, *IRR* Incidence rate ratio, *n.c*. Not calculable, *SLE* Systemic lupus erythematosus

^a^Adjusted for age, sex, socioeconomic status, and geographic region

### Risk of developing other SLE-related ophthalmic disorders in patients with SLE

The incidence rates and IRRs for developing other important SLE-related ophthalmic disorders, including episcleritis and scleritis, retinal vascular occlusion, retinal vasculitis, and neovascular glaucoma [2] in the SLE cohort and the comparison cohort are shown in Table 6. Overall, patients in the SLE cohort exhibited a significantly higher risk of developing episcleritis and scleritis (adjusted IRR 6.11, *P* <  0.001) and retinal vascular occlusion (adjusted IRR 3.18, *P* = 0.023) compared with those in the comparison cohort. There is a statistical trend that patients in the SLE cohort might have a higher risk of developing retinal vasculitis (adjusted IRR 12.00, *P* = 0.079). However, the IRR was not calculable for neovascular glaucoma due to no cases were identified in the comparison cohort.

**Table 6:** The incidence rate and incidence rate ratio of episcleritis and scleritis, retinal vascular occlusion, retinal vasculitis, and neovascular glaucoma in the systemic lupus erythematosus cohort and comparison cohort

Disorder	SLE cohort			comparison cohort			IRR (95% CI)	Adjusted IRR^a^ (95% CI)
(ICD-9-CM)	No. of patient	Person-years	IR	No. of patient	Person-years	IR	*P*	*P*
Episcleritis and scleritis (379.0x) [*N* = 5715]	8	3186	2.51	13	34,044	0.38	6.57 (2.72–15.86) < 0.001	6.11 (2.39–15.62) < 0.001
Retinal vascular occlusion (362.3x) [*N* = 5723]	4	3197	1.25	11	34,077	0.32	3.88 (1.23–12.17) 0.020	3.81 (1.21–12.03) 0.023
Retinal vasculitis (362.18) [*N* = 5731]	1	3215	0.31	1	34,163	0.03	10.63 (0.66–169.89) 0.095	12.00 (0.75–192.41) 0.079
Neovascular glaucoma (365.63) [*N* = 5730]	1	3218	0.31	0	34,151	n. c.	n. c.	n. c.

*CI* Confidence interval, *ICD-9-CM* International Classification of Diseases, Ninth revision, clinical modification, *IR* Incidence rate per 1000 person-years, *IRR* Incidence rate ratio, *n.c*. Not calculable, *SLE* Systemic lupus erythematosus*.*

^a^Adjusted for age, sex, socioeconomic status, and geographic region

## Discussion

Our population-based cohort study showed that patients with SLE had a higher prevalence and frequency of outpatient ophthalmologist visits compared with patients without SLE. These findings are consistent with previous research that SLE could attack the ocular system. However, previous studies on SLE with ophthalmic involvement focused mainly on severe eye manifestations, such as retinal necrosis and vaso-occlusive disease that can lead to visual impairment [2, 3]. In contrast, our study showed the comprehensive impact of SLE on the ocular system.

We observed that the overall proportion of outpatient ophthalmic visits was higher in patients with SLE (68.1% vs. 60.5%), the mean and median frequencies of visits were both significantly elevated. This observation shows that ophthalmic disorders were highly common in patients with SLE. Among the common ophthalmic disorders for outpatient ophthalmologist visits in patients with SLE, the proportion and frequency of visits for chronic conjunctivitis, dry eye syndrome, acute and chronic allergic conjunctivitis, keratitis, refractive disorders, acute conjunctivitis, cataracts, and retina disorders were significantly higher in patients with SLE compared with those without SLE. A possible reason for the increase in the refractive disorders might be due to myopia, which is caused by changes in the curvature or refractive index of the lens or the anterior shift of the iris-lens diaphragm as a result of SLE-related inflammation in the nearby tissue [14, 15]. Animal models have demonstrated that chronic inflammation could lead to the development of myopia [16]. For allergic conjunctivitis, a recent study showed that atopic diseases, including allergic conjunctivitis was strongly associated with SLE [17]. However, to the best of our knowledge, this study is the first to show that patients with SLE had increased frequency and proportion of developing acute conjunctivitis, which is a disease due to bacterial or viral infection of the conjunctiva. It is reasonable to expect the risk of bacterial and viral infection could be increased due to impaired immunity in patients with SLE. SLE is known to cause retinal vasculitis, which can lead to vessel occlusion, vitreous hemorrhage, retinal traction, and retinal detachment. This will result in lupus retinopathy [7], a marker of poor prognosis for survival [18, 19].

Dry eye syndrome is very common in patients with SLE owing to the inflammation of the lacrimal glands [20]. As expected, we also observed a significantly higher frequency and proportion of dry eye syndrome in patients with SLE. Moreover, the frequency and proportion of common consequences of dry eyes, including chronic conjunctivitis and keratitis, as a result of SLE-related lacrimal gland manifestations, were also significantly elevated among patients with SLE in this study. Results from our sub-group analyses further showed that the risk of developing dry eye syndrome was significantly elevated in patients with SLE, regardless of sex or age group.

As for cataracts and glaucoma, it has been reported that patients with SLE have a higher prevalence of both cataracts and glaucoma because of long-term steroid use [21, 22]. We observed that the frequency and proportion of visits for cataracts were significantly higher, but for glaucoma, only a statistical trend was observed. Nevertheless, after controlling for sex, age, socioeconomic status, and geographic region, the adjusted IRR were significantly higher for both cataracts and glaucoma in patients with SLE. In addition, there were some differences in the risk between the sexes. For cataracts, the risk was significantly elevated in both sexes, with a higher adjusted IRR in female patients with SLE. While the risk was significantly elevated in both sexes for glaucoma, with a higher risk observed in male patients with SLE. For both cataracts and glaucoma, the risk was highest among patients with SLE in the youngest age group. It is worth noting that pediatric patients with SLE suffer from a higher prevalence of glaucoma and cataracts [23]. Furthermore, POAG has previously been suggested to relate to neuroendocrine-immune abnormalities [12]. The present study was not able to demonstrate a significant difference in the prevalence of POAG between the SLE and comparison cohort. Nevertheless, despite the use of a population-based database in this study, the observed number of POAG is still small. The role of immune system in POAG will require further studies to elucidate.

In this study, we found that the risk of developing several rare SLE-related ophthalmic diseases, including episcleritis and scleritis, and retinal vascular occlusion were elevated in patients with SLE. Episcleritis and scleritis were previously noted to be associated with several systemic autoimmune diseases [24]. The development of episcleritis and scleritis in patients with SLE might be attributed to the deposition of immunoglobulins [25]. Retinal vascular occlusion is a serious ophthalmic condition, which could decrease visual acuity in patients with SLE. The development of retinal vascular occlusion is related to the presence of antiphopholipid antibodies and elevated disease activity in patient with SLE [26, 27].

A few study limitations should be mentioned. First, serological and clinical data were not available for analysis, which is a constraint of our claim-based data source. Second, the possibility of misclassification could not be completely ruled out because all diagnoses were based on the administrative data. Nevertheless, the Bureau of National Health Insurance routinely conducts audits of patient records to improve accuracy. Third, the sample size for male patients with SLE was relatively small, which precluded further analyses of ophthalmic disorders with low prevalence. Fourth, the prevalence of some SLE-related ophthalmic disorders, such as retina vasculitis and neovascular glaucoma was too low for analysis. Fifth, mild cases of chronic conjunctivitis, dry eye syndrome, cataracts, and refractive disorders could be underestimated because they might not necessary result in a medical visit.

## Conclusions

This secondary cohort analysis of a population-based health claims database in Taiwan showed that patients with SLE were associated with a high utilization of outpatient ophthalmic medical visits. A high proportion and frequency of ophthalmologist visits for chronic conjunctivitis, dry eye syndrome, allergic conjunctivitis, keratitis, refractive disorders, acute conjunctivitis, cataracts, and retina disorders were observed in both male and female patients with SLE. The significantly increased incidence of dry eye disease, cataracts, glaucoma, episcleritis and scleritis, and retinal vascular occlusion in patients with SLE deserves a high level of vigilance by clinicians.

## Data Availability

The data are not publicly available due to the Taiwan Personal Information Protection Act. The data that support the findings of this study are available on request from the corresponding author.

## Abbreviations

CI: Confidence interval
ICD-9-CM: International Classification of Diseases, 9th revision, Clinical Modification Declarations
IR: Incidence rate
IRR: Incidence rate ratio
LHID: Longitudinal Health Insurance Database
NHIRD: National Health Insurance Research Database
SLE: Systemic lupus erythematosus
